# Prevalence and predictors of kinesiophobia in psoriatic arthritis: the role of central sensitization and comorbidities

**DOI:** 10.3389/fmed.2026.1801448

**Published:** 2026-03-24

**Authors:** Rocío Llamas-Ramos, Inés Llamas-Ramos, Jorge Juan Alvarado-Omenat, Esther Toledano, Rubén Queiró, María José Fernández-Gómez, Javier Martín-Vallejo, Carolina Cristina Chacón, Roberto Díaz-Peña, Daniel Martín, Cristina Hidalgo, María Dolores Sánchez, Carlos Montilla

**Affiliations:** 1 Department of Nursing and Physiotherapy, Universidad de Salamanca, Salamanca, Spain; 2 Institute of Biomedical Research of Salamanca (IBSAL), Salamanca, Spain; 3 Hospital of Salamanca, Salamanca, Spain; 4 FisioSport Salamanca, Salamanca, Spain; 5 Department of Rheumatology, San Carlos Clinical Hospital, Madrid, Spain; 6 Department of Rheumatology, Hospital Universitario Central de Asturias (HUCA), Oviedo, Spain; 7 Department of Statistics, Universidad de Salamanca, Salamanca, Spain; 8 Department of Rheumatology, Clinical University Hospital of Salamanca, Salamanca, Spain; 9 Immunogenetics Lab, Fundación Pública Galega de Medicina Xenómica, SERGAS, Grupo de Medicina Xenómica-USC,Instituto de investigación Sanitaria de Santiago de Compostela (IDIS), Santiago de Compostela, Spain; 10 Faculty of Health Sciences, Universidad Autónoma de Chile, Talca, Chile; 11 Primary Care Management Valladolid West, Valladolid, Spain; 12 Department of Rheumatology, Hospital de Zamora, Zamora, Spain; 13 Department of Medicine, University of Salamanca (USAL), Salamanca, Spain

**Keywords:** central sensitization, disability, fear-avoidance, kinesiophobia, pain amplification, psoriatic arthritis, sleep quality

## Abstract

**Background:**

Kinesiophobia (excessive fear of movement due to belief of injury) is highly prevalent in rheumatologic diseases, yet its prevalence and associations in psoriatic arthritis (PsA) remain unexplored.

**Objective:**

To determine the prevalence of kinesiophobia in PsA and examine its associations with central sensitization (CS), demographic characteristics, disease activity, physical function, and patient-reported outcomes.

**Methods:**

Cross-sectional study of 246 consecutive PsA patients. Kinesiophobia was assessed using the Tampa Scale of Kinesiophobia-11. CS was measured by Central Sensitization Inventory (CSI). Disease activity, functional status, physical activity, sleep quality, anxiety, depression, and fatigue were systematically evaluated. Univariate and multivariable analyses (logistic and linear regression) were performed.

**Results:**

Kinesiophobia was present in 45.5% (112/246) of patients. Patients with kinesiophobia demonstrated significantly higher CSI scores (41.5 vs. 29; *p* < 0.001), reduced physical activity (1619.5 vs. 2,970 MET-minutes/week; *p* = 0.01), greater disease activity (cDAPSA: 13 vs. 11; *p* = 0.001), functional impairment (HAQ-DI; *p* = 0.001), and increased comorbid anxiety and depression (*p* = 0.001). A significant correlation existed between kinesiophobia and CSI (r = 0.39; *p* < 0.001). In multivariable logistic regression, central sensitization (OR: 1.03; 95% CI: 1.00–1.05; *p* = 0.02) and sleep quality (PSQI; OR: 1.09; 95% CI: 1.00–1.1; *p* = 0.03) emerged as independent predictors, explaining 20% of kinesiophobia variance. In linear regression, these variables accounted for 12% of variance (R^2^ = 0.12).

**Conclusion:**

Kinesiophobia functions as an amplifier of pain perception and functional disability, particularly in patients with symptom-inflammation discordance. A bidirectional pathophysiologic relationship between kinesiophobia and CS likely perpetuates chronic pain and disability. Multidimensional interventions may enhance clinical outcomes in kinesiophobic PsA patients, especially those with high perceived impact despite adequate inflammatory control.

## 1 Introduction

Psoriatic arthritis (PsA) is an immune-mediated systemic inflammatory disease characterized by inflammation of synovial joints and/or entheses (1, 2). The primary clinical manifestation of PsA is pain related to articular or entheseal inflammation. Despite significant therapeutic advances over recent decades, reduction in inflammatory markers has not consistently correlated with improvements in pain intensity in a substantial proportion of PsA patients (3). This phenomenon has been linked to central sensitization (CS), a neurobiological state characterized by amplified nociceptive signal processing in the central nervous system (4).

Chronic pain perpetuation in PsA can lead to fear of movement and activity avoidance, a process termed kinesiophobia, defined as excessive, irrational, and debilitating fear of movement or physical activity resulting from the catastrophic belief that movement will cause pain, injury, or re-injury (5, 6). Kinesiophobia is grounded in the fear-avoidance model (FAM) proposed by Vlaeyen and Linton, which posits that catastrophic pain interpretation leads to movement fear, which drives avoidance behaviors (7, 8). Sustained avoidance results in functional loss, increased disability perception, and paradoxically, pain intensification, perpetuating a vicious cycle that aggravates functional limitation (7, 8). Importantly, kinesiophobia may serve as a critical mediator amplifying the relationship between inflammatory disease activity, central pain processing abnormalities, and functional disability—particularly in PsA patients experiencing a discordance between objective inflammatory markers and symptom severity.

Kinesiophobia is highly prevalent across numerous inflammatory rheumatic diseases. In rheumatoid arthritis (RA), recent studies document a prevalence of 70.86%—markedly elevated compared to 12% in healthy controls (9). Kinesiophobia in RA patients correlates significantly with greater disease activity, functional deterioration, disability, increased pain intensity, and reduced physical activity levels (9, 10). In axial spondyloarthritis (axSpA), kinesiophobia prevalence ranges from 35 to 50% (11). A 2025 study of 28 axSpA patients demonstrated that perceived pain, pain catastrophizing, and fear-avoidance beliefs explain substantial variance in disease activity (BASDAI) and functionality (BASFI) (12). In gout, kinesiophobia prevalence reaches 62.3%, with moderate-to-strong positive correlations between TSK (Tampa Scale of Kinesiophobia) and CSI scores (*ρ* = 0.650; *p* < 0.001) (13).

A bidirectional relationship between kinesiophobia and central sensitization is well-documented: CS patients experience amplified and generalized pain perception, which magnifies catastrophic movement interpretation as threatening, facilitating kinesiophobia development and maintenance (14, 15); conversely, sustained avoidance behaviors from kinesiophobia generate neuroplastic changes contributing to central sensitization, with physical inactivity and chronic muscle tension from movement fear perpetuating nociceptive hypersensitivity (16, 17).

Despite the relationship between kinesiophobia and other rheumatic diseases affecting the joints, such as rheumatoid arthritis and axial spondyloarthritis, having been described, the higher burden of emotional comorbidities in psoriatic arthritis, which may contribute to the discordance between inflammation and pain, makes the study of kinesiophobia in this disease particularly relevant. Therefore, this study aimed to determine kinesiophobia prevalence in a PsA cohort and examine associations with central sensitization, demographic characteristics, disease activity, patient-reported outcomes (pain, anxiety, depression), and physical function measures (functional disability, physical activity).

## 2 Methods

### 2.1 Study design and ethical approval

A single-center cross-sectional observational study was conducted from November 1, 2024, to October 24, 2025. The study received approval from the Ethics Committee of Salamanca University Hospital Complex (Code CEIC: PI 2024 10 1747-TFG) and was conducted according to Helsinki Declaration principles and current biomedical research regulations.

### 2.2 Study population

Consecutive patients diagnosed with PsA according to CASPAR criteria (18) who consented to participate and provided informed consent were recruited. Inclusion criteria: confirmed PsA diagnosis, age ≥18 years, and capacity to comprehend and complete questionnaires. Exclusion criteria: cognitive or communication difficulties preventing adequate instrument completion, refusal to participate, or incomplete data collection.

### 2.3 Variables and measurement instruments

#### 2.3.1 Baseline variables

Age, sex, disease duration (years), smoking status (current, former, never), and clinical PsA presentation (peripheral: peripheral joint pain/swelling; axial: inflammatory spinal pain with radiographic sacroiliitis grade II + or syndesmophytes; mixed: both features) were documented. Disease-modifying antirheumatic drug (DMARD) use was categorized: conventional synthetic (csDMARDs), biologic (bDMARDs), and targeted synthetic (tsDMARDs). Number of patients refractory to ≥2 bDMARDs/tsDMARDs, enthesial sites affected (modified Maastricht Ankylosing Spondylitis Enthesitis Score—mMASES; range 0–15) (19), dactylitis history (present/past), and skin involvement (Psoriasis Area and Severity Index—PASI) were recorded (20).

Physical activity was measured using the International Physical Activity Questionnaire (IPAQ) (21). The questionnaire assesses intensity (low, moderate, vigorous), frequency (days/week), and duration (minutes/day) of activity, with results expressed in metabolic equivalent of task (MET) minutes/week. One MET represents resting energy expenditure (~3.5 mL O₂/kg/min or 1 kcal/kg/h). METs were estimated by multiplying MET scores (low: 3.3; moderate: 4.0; vigorous: 8.0) by days/week and minutes/day of activity.

##### 2.3.1.1 Kinesiophobia assessment

Kinesiophobia was measured using the Tampa Scale of Kinesiophobia-11 (TSK-11), an 11-item questionnaire with four response options reflecting kinesiophobia intensity (22). Final scores range 11–44 points, with higher scores indicating greater kinesiophobia. A TSK-11 score >26 was considered indicative of clinically significant kinesiophobia based on recent validation studies (9, 23). In our study, we adopted this threshold because it has been proposed and validated as a clinically relevant cut-off for kinesiophobia severity in chronic musculoskeletal pain populations and has been used in recent rheumatologic cohorts, thereby facilitating comparability with previous literature (9, 23). Nevertheless, we acknowledge that this represents a generic chronic pain threshold rather than a PsA-specific value. We selected this reduced version because it has psychometric properties comparable to those of the original 17-item Tampa Scale, while being briefer and more feasible for routine clinical use (22).

##### 2.3.1.2 Central sensitization assessment

Central sensitization was measured using the Central Sensitization Inventory (CSI), Part A, comprising 25 items rated on a 5-point Likert scale (0 = “never” to 4 = “always”), with total score range 0–100. CSI ≥ 40 was classified as central sensitization present. CS levels were categorized as: subclinical (0–29), mild (30–39), moderate (40–49), severe (50–59), and excessive (≥60). CSI Part B assessed presence of 10 central sensitivity syndromes (restless leg syndrome, chronic fatigue syndrome, fibromyalgia, temporomandibular joint disorder, migraines, irritable bowel syndrome, multiple chemical sensitivities, cervical trauma, panic/anxiety attacks, depression) (24).

#### 2.3.2 Disease activity and functionality measures

##### 2.3.2.1 Disease activity

In peripheral PsA patients, disease activity was measured using the clinical Disease Activity Index for Psoriatic Arthritis (cDAPSA), calculated by summing tender joint count (0–68), swollen joint count (0–66), patient global assessment score (0–10 numerical rating scale [NRS]), and pain NRS (0–10) (25). For axial manifestations, disease activity was measured using ASDAS-CRP (Ankylosing Spondylitis Disease Activity Score with C-reactive protein) (26).

##### 2.3.2.2 Functionality

Physical function was assessed using the Health Assessment Questionnaire-Disability Index (HAQ-DI), a self-administered questionnaire measuring ability to perform activities of daily living (dressing, walking, etc.) (27). In patients with axial manifestations, functional limitations were additionally evaluated with the Bath Ankylosing Spondylitis Functional Index (BASFI), a 10-item instrument that assesses the impact of axial disease on everyday tasks such as bending, reaching and standing (28).

Disease Impact: Disease impact was obtained via Psoriatic Arthritis Impact of Disease (PsAID-12), which measures clinical manifestations (pain, skin sensations, fatigue, work/leisure limitations, function, discomfort, sleep, coping) and comorbidities (anxiety, embarrassment, social life, depression) (29). The PsAID-12 comprises 12 items rated 0–10 NRS.

#### 2.3.3 Comorbidity assessment

##### 2.3.3.1 Fatigue

Assessed using the Functional Assessment of Chronic Illness Therapy (FACIT-Fatigue) scale, validated for PsA. The 13-item questionnaire, rated on a 5-point Likert scale (0–4), yields total scores 0–52 (higher scores indicating less fatigue) (30).

##### 2.3.3.2 Anxiety and depression

Hospital Anxiety and Depression Scale (HADS), a 14-item scale identifying anxiety/depression in medical conditions. Subscale scores range 0–21 (HADS-Anxiety [HADS-A], HADS-Depression [HADS-D]) and classify as: normal (0–7), borderline abnormal (8–10), or abnormal (11–21) (31).

##### 2.3.3.3 Sleep quality

Pittsburgh Sleep Quality Index (PSQI), a 19-item self-report assessing sleep quality over 30 days. PSQI explores seven domains (subjective sleep quality, sleep latency, sleep duration, habitual sleep efficiency, sleep disturbances, sleep medication use, daytime dysfunction), each scored 0–3, with global score range 0–21. PSQI ≥6 indicates poor sleep quality (32).

##### 2.3.3.4 Obesity

Body mass index (BMI; kg/m^2^) was calculated as weight (kg)/height^2^ (m^2^) (33).

##### 2.3.3.5 Fibromyalgia

Fibromyalgia presence was assessed using 2016 criteria (34).

### 2.4 Procedure

After participant inclusion, all variables were collected in a single visit via self-administered questionnaires and medical chart review. Trained professionals performed evaluations following standardized protocols.

### 2.5 Statistical analysis

Quantitative variables were expressed as means ± standard deviation (SD) for normally distributed variables and as median (interquartile range [IQR]) for non-normally distributed variables. Qualitative variables were presented as numbers and percentages.

Between-group comparisons utilized Student’s *t*-test for normally distributed quantitative variables and Mann–Whitney U test for ordinally/non-normally distributed variables. Comparisons among >2 groups used one-way ANOVA (normally distributed) or Kruskal-Wallis H test (ordinal/non-normally distributed). Correlations between quantitative variables were assessed using Spearman’s method (35).

A multiple linear regression analysis was conducted with kinesiophobia as the dependent variable and statistically significant univariate variables with potential kinesiophobia effects as independent variables, complemented by variables previously associated with kinesiophobia in the literature. Logistic regression was also used to determine which variables were possible predictors of the presence of kinesiophobia. Model goodness-of-fit was assessed using the adjusted R-Square for linear regression and the Nagelkerke R^2^ for logistic regression. Both models were carried out exclusively to explore the effect of independent variables on kinesiophobia and therefore their value is purely exploratory.

The statistical power for multiple regression was 99% for 12 independent variables, a significance level of 0.05, an effect size of 0.15, and a sample size of 246. To analyze the reliability and stability of the logistic regression results, the sample size was calculated based on the events per variable, the number of predictor variables, and the expected proportion (36). If 10 events per variable, 11 independent variables, and an expected proportion of 0.5 are established, the sample size would be 220.

Statistical significance was set at *p* < 0.05. Analysis was performed using IBM SPSS Statistics for Windows, Version 25.0. The “powermediation v. 0.3.4” function from R statistics v.4.5.0 was used to calculate the power of the models.

## 3 Results

### 3.1 Baseline characteristics

A total of 246 PsA patients were enrolled (median age 55 years [IQR: 12]; 139 males [56.5%]). Kinesiophobia was present in 112 patients (45.5%). Baseline variables and their relationship with kinesiophobia presence are summarized in Table 1.

**Table 1:** General characteristics and kinesiophobia associations.

Variable	All (*n* = 246)	Kinesiophobia (*n* = 112)	No kinesiophobia (*n* = 134)	*p*
Age	55 (12)	55 (12)	55 (13)	0.8
Sex (male/female)	139/107	57/55	82/52	0.1
Disease duration (years)	9 (8)	10 (8)	8 (9)	0.2
Smoking status, *N* (%)				0.6
Smoker	67 (27)	29 (25.9)	38 (28.4)	
Former smoker	107 (44)	52 (46.4)	55 (51.4)	
Non smoker	72 (29)	31 (27.7)	41 (30.6)	
Conventional synthetic DMARDs, *N* (%)	157 (63.8)	74 (47.1)	83 (52.9)	0.8
Methotrexate	120 (77)	58 (78.4)	62 (74.7)	
Sulfasalazine	27 (17)	12 (16.2)	15 (18.1)	
Leflunomide	10 (6)	4 (5.4)	6 (7.2)	
tsDMARDs or bDMARDs, *N* (%)	79 (32.1)	45 (57)	34 (43)	0.01
TNFα inhibitor	51 (70)	29 (64.5)	22 (64.7)	
Secukinumab	12 (16)	6 (13.4)	6 (17.6)	
Ustekinumab	6 (8)	5 (11.1)	1 (2.9)	
Guselkumab	4 (6)	2 (4.4)	2 (6)	
Tofacitinib	5 (6)	2 (4.4)	3 (8.9)	
Apremilast	1 (6)	1 (2.2)	0 (0)	
Clinical presentation, *N* (%)				0.8
Peripheral	215 (87.4)	99 (88.4)	116 (86.6)	
Mixed	28 (11.4)	12 (10.7)	16 (12)	
Axial	3 (1.2)	1 (0.9)	2 (1.4)	
Dactylitis (presente o pasada) *N* (%)	51 (20.7)	28 (25)	23 (17.2)	0.1
mMASES	0 (2)	1 (3)	0 (2)	0.001
PASI	0.4 (2.6)	1 (3)	0 (2)	0.2
Physical activity (MET minutes/week)	2,613 (6782.2)	1619.5 (5779.7)	2,970 (8475.5)	0.01
CSI	34.4 (22)	41.5 (21)	29 (21)	0.001

DMARD, disease-modifying antirheumatic drug; tsDMARD, targeted synthetic disease-modifying antirheumatic drug; bDMARD, biologic disease-modifying antirheumatic drug; mMASES, modified Maastricht Ankylosing Spondylitis Enthesitis Score; PASI, Psoriasis Area Severity Index. CSI, Central Sensitization Inventory; MET, Metabolic Equivalent of Task.

Data revealed significant kinesiophobia-CSI correlation (r = 0.39; *p* < 0.001). Differences were observed regarding bDMARD/tsDMARD use (27 [10] vs. 24.1 [12]; *p* = 0.04), enthesial sites affected (r = 0.18; *p* = 0.006), and physical activity (r = −0.17; *p* = 0.01).

No correlations were found with age (r = −0.27; *p* = 0.6) or disease duration (r = 0.0; *p* = 0.1). No differences occurred with sex (M/F: TSK-11 23.7 [IQR: 12] vs. 26 [IQR: 12]; *p* = 0.1), smoking status (r = 0.5), clinical presentation (*p* = 0.6), or dactylitis presence (*p* = 0.3).

### 3.2 Association of kinesiophobia with disease activity, pain, functionality, and disease impact

Patients with kinesiophobia showed higher clinical disease activity, more closely related to pain than to inflammatory parameters. Notably, kinesiophobia demonstrated a significant correlation with pain NRS (r = 0.24; *p* < 0.001) and cDAPSA (r = 0.25; *p* < 0.001), but no correlation with CRP (r = 0.08; *p* = 0.2) The results are presented in Table 2.

**Table 2:** Activity, pain, functionality, and disease impact measures.

Variable	All (*n* = 246)	Kinesiophobia (*n* = 112)	No kinesiophobia (*n* = 134)	*P*
cDAPSA*	11 (9.2)	13 (9)	10 (11)	0.001
Activity VAS	4 (4)	5 (4)	4 (5)	0.001
Pain VAS	4 (4)	5 (4.7)	8 (9)	0.001
TJC	1 (2)	2 (2)	1 (2)	0.001
SJC	1 (1)	1 (1)	1 (1.2)	0.3
CRP (mg/dL)	0.2 (0.4)	0.2 (0.4)	0.2 (0.4)	0.4
ASDAS PCR**	2.1 (SD:0.8)	2.2 (SD:0.9)	2 (SD:0.7)	0.4
HAQ-DI*	0.5 (0.8)	0.6 (0.7)	0.3 (0.8)	0.001
BASFI**	2.5 (4.3)	2.9 (4.3)	2.1 (4.9)	0.5
PSAID	3.2 (3.5)	3.9 (3.3)	2.4 (3.3)	0.001

cDAPSA, Clinical Disease Activity Index for Psoriatic Arthritis; VAS, visual analogue scale; TJC, tender joint count; SJC, swollen joint count; CRP, C-reactive protein; ASDAS-CRP, Ankylosing Spondylitis Disease Activity Score with C-reactive protein; HAQ-DI, Health Assessment Questionnaire-Disability Index; BASFI, Bath Ankylosing Spondylitis Functional Index; PsAID, Psoriatic Arthritis Impact of Disease. * In peripheral and mixed forms (*n* = 243). ** In axial and mixed forms (*n* = 31).

cDAPSA correlated with kinesiophobia (r = 0.25; *p* < 0.001), including pain NRS (r = 0.24; *p* < 0.001) and activity NRS (r = 0.23; *p* < 0.001). HAQ-DI (r = 0.28; *p* < 0.001) and PSAID (r = 0.36; *p* < 0.001) showed significant correlations; BASFI did not (r = 0.0; *p* = 0.7).

### 3.3 Relationship of kinesiophobia with comorbidities

Kinesiophobia was associated with fatigue, HADS-A, HADS-D, and PSQI. The results are presented in Table 3.

**Table 3:** Comorbidity measures.

Variable	All (*n* = 246)	Kinesiophobia (*n* = 112)	No kinesiophobia (*n* = 134)	*P*
FACIT-F	38 (17)	34 (15)	42 (16)	0.001
HADS-A	5 (6)	6.4 (6)	5 (5)	0.001
HADS-D	4 (6)	5.2 (5)	3 (5)	0.001
PSQI	7.4 (8)	10.3 (8)	6 (6)	0.001
BMI (kg/m^2^)	27.1 (SD: 4,5)	27.4 (SD:4.8)	26.6 (SD:4.3)	0.4
Fibromyalgia *N* (%)	16 (6.5)	12 (10.7)	4 (3)	0.01

FACIT-F, Functional Assessment of Chronic Illness—Fatigue; HADS-A and HADS-D, Hospital Anxiety and Depression Scale Anxiety and Depression subscales, respectively; PSQI, Pittsburgh Sleep Quality Index; BMI, body mass index.

The FACIT-Fatigue score correlated with kinesiophobia (r = −0.25; *p* < 0.001), as did HADS-Anxiety (r = 0.24; *p* < 0.001), HADS-Depression (r = 0.27; *p* < 0.001), PSQI (r = 0.34; *p* < 0.001), and fibromyalgia presence (28.6 [IQR: 10] vs. 25.0 [IQR: 12]; *p* = 0.02). No correlation was found with BMI (r = 0.09; *p* = 0.1).

In the logistic regression analysis, using kinesiophobia as the dependent variable (present/absent) and sex, SCI, MASES, IPAQ, TJC, VAS pain, FACIT-f, HADS-A, HADS-D, PSQI, and presence of fibromyalgia as independent variables, we obtained the following results: SCI: OR: 1.03; *p* = 0.02; 95% CI: (1.00–1.05) and PSQI: OR: 1.09; *p* = 0.03; 95% CI: (1.00–1.10). The R2 Nagelkerke was 0.20. No significant association was found with: MASES (*p* = 0.7), IPAQ (*p* = 0.1), NAD (*p* = 0.1), VAS pain (*p* = 0.1), FACIT-f (*p* = 0.5), HADS-A (*p* = 0.2), HADS-D (*p* = 0.4), and presence of fibromyalgia (*p* = 0.6).

In the linear regression analysis using kinesiophobia as the dependent variable and sex, SCI, MASES, IPAQ, TSC, VAS pain, FACIT-f, HADS-A, HADS-D, PSQI, and presence of fibromyalgia as independent variables, we obtained: SCI: *β*: 0.14; *p* < 0.001; 95% CI: (0.05–0.22) and PSQI; β: 0.30; *p* = 0.02; 95% CI: (0.03–0.57). The adjusted R- Squares was: 0.12. No significant association was found with: sex (*p* = 0.5), MASES (*p* = 0.4), IPAQ (*p* = 0.5), NAD (*p* = 0.4), VAS pain (*p* = 0.4), FACIT-f (*p* = 0.4), HADS-A (*p* = 0.5), HADS-D (*p* = 0.4), and presence of fibromyalgia (*p* = 0.9).

## 4 Discussion

This is the first study to specifically examine the prevalence of kinesiophobia and its associations with clinical, demographic, and psychosocial variables in a cohort of patients with PsA. However, the associations detected should be interpreted with great caution without attributing them a causal nature, given the type of sample and the purely exploratory nature of the models developed. Our findings reveal that kinesiophobia is highly prevalent in this population (45.5%), confirming the importance of recognizing and addressing this phenomenon in clinical practice. The prevalence of kinesiophobia in our study places PsA between prevalence rates reported in other inflammatory rheumatic diseases but with distinctive characteristics. In patients with rheumatoid arthritis (RA), recent studies report a prevalence of 70.86%—substantially higher than the 12% reported in healthy controls (9, 10). In axial spondyloarthritis (axSpA), prevalence ranges from 35 to 50% (11), whereas gout demonstrates a documented prevalence of 62.3% (13). This variability in prevalence rates may be attributed to the heterogeneous clinical phenotype of PsA, characterized by predominantly peripheral involvement (87.4% of patients in our study), contrasting with the predominance of axial manifestations in spondyloarthropathies. Second, pain in PsA is fundamentally articular, whereas in axSpA inflammatory spinal pain may generate greater movement avoidance. Furthermore, the magnitude of central sensitization, as reflected in significantly higher CSI scores in kinesiophobic patients (mean CSI: 41.5 vs. 29; *p* < 0.001), may differentially modulate fear-of-movement responses across diagnoses.

One of the most relevant findings in our study is the significant correlation between kinesiophobia and CS (r: 0.39; *p* < 0.001). This association is not purely correlative but may reflect a bidirectional pathogenic mechanism well documented in the literature (14, 15). When patients experience CS, pain perception amplifies and generalizes, causing normal or subliminal stimuli to be interpreted as threatening (37). This nociceptive hyperexcitability may amplify catastrophic movement interpretation as potentially harmful and has been hypothesized to be associated with kinesiophobia development and maintenance. Conversely, sustained avoidance behaviors generated by kinesiophobia have been proposed to contribute to neuroplastic changes. Prolonged physical inactivity, chronic muscle tension resulting from movement fear, and diminished proprioceptive input may contribute to a state of central nociceptive hypersensitivity (38). This cycle is particularly relevant in PsA, where pre-existing articular inflammation already predisposes to central pain processing changes (16). In our logistic regression analysis, central sensitization emerged as a significant independent variable associated with kinesiophobia (OR: 1.03; *p* = 0.02; 95% CI: 1.00–1.05), although it explained only 20% of variance (R^2^ = 0.20). Rather than representing a limitation, this relatively low R^2^ is itself a substantive finding: kinesiophobia is not explained solely by pain severity, physical activity levels, or classical psychosocial comorbidities. This observation reinforces the biopsychosocial nature of kinesiophobia and suggests that other important determinants—possibly self-efficacy beliefs, social support, prior trauma experiences, or genetic factors (38, 39)—remain unevaluated (39, 40).

The number of affected enthesial sites showed positive correlation with kinesiophobia (r: 0.18; *p* = 0.006). Enthesitis in PsA is a characteristic manifestation generating focal pain, particularly at weight-bearing sites such as the Achilles tendon insertion and plantar fascia (41). Direct palpation pain at these sites, aggravated by physical activity, may be associated with anticipatory fear of movement and weight-bearing activities, potentially contributing to an avoidance cycle.

Kinesiophobic patients demonstrated higher cDAPSA scores (13 vs. 11; *p* = 0.001), including elevated pain NRS (5 vs. 4; *p* = 0.001) and greater perceived activity impact (activity NRS: 5 vs. 4; *p* = 0.001). Significant correlations with total cDAPSA (r: 0.25; *p* < 0.001) and individual components such as pain NRS (r: 0.24; *p* < 0.001) and activity NRS (r: 0.23; *p* < 0.001) suggest a tight linkage between movement fear and disease activity perception (42).

Patients with higher TSK-11 scores (indicating greater kinesiophobia) showed a greater proportion receiving bDMARDs or tsDMARDs (univariate analysis OR). This association warrants cautious interpretation and does not suggest that biologic therapy causes kinesiophobia. Rather, this reflects confounding by indication: patients with more active disease require biologic therapy and may simultaneously experience greater kinesiophobia due to underlying higher inflammatory activity and pain burden. Notably, bDMARD/tsDMARD status did not remain significant in multivariable analysis, reinforcing that this parameter is driven by the confounding relationship between disease activity, pain, and treatment intensity rather than a direct effect of biologic agents.

Higher kinesiophobia scores demonstrated significantly reduced physical activity (1619.5 vs. 2,970 MET-minutes/week; *p* = 0.01). While this association is conceptually expected given avoidance behaviors, it is important to emphasize that causality cannot be established in this cross-sectional design. It is plausible that both kinesiophobia and physical inactivity are consequences of an amplified chronic pain state rather than direct causal relationships. Similarly, the association with HAQ-DI (r: 0.28; *p* < 0.001) is particularly clinically important, as the HAQ reflects functional disability in activities of daily living (27). Kinesiophobic patients reported greater difficulty dressing, ambulating, and performing daily tasks, reinforcing that kinesiophobia transcends psychological fear and impacts concrete physical functionality.

The correlation with FACIT-Fatigue was significant (r: −0.28; *p* < 0.001), indicating greater fatigue in kinesiophobic patients. Fatigue in PsA is multifactorial, derived from systemic inflammation, central pain processing changes, and psychosocial factors such as depression and sleep disturbance (43). Movement fear and physical inactivity may be associated with greater fatigue through cardiovascular and muscular deconditioning, potentially perpetuating a vicious cycle.

Kinesiophobic patients demonstrated significantly elevated HADS-Anxiety (6.4 vs. 5; *p* = 0.001) and HADS-Depression (5.2 vs. 3; *p* = 0.001) scores. Correlations were moderate (r: 0.24 for anxiety, r: 0.27 for depression; both *p* < 0.001). This association is bidirectional: anxiety and depression amplify pain catastrophizing and movement fear, while sustained kinesiophobia-driven activity avoidance limits social and physical activity—protective mental health factors (44).

In the logistic regression analysis, sleep quality (PSQI) emerged as a significant independent predictor of kinesiophobia (OR: 1.09; *p* = 0.03; 95% CI: 1.00–1.1), indicating that sleep disturbance is an important clinical factor. Lack of restorative sleep has been shown to amplify pain sensitivity and reduce coping capacity, which may in turn be associated with movement fear development (45).

Although the number of fibromyalgia patients was limited (*n* = 16, 6.5%), they demonstrated elevated kinesiophobia levels (28.6 vs. 25; *p* = 0.02). This finding is expected and consistent with literature, as fibromyalgia represents an amplified central sensitization state—a determining factor for kinesiophobia (46, 47).

Interestingly, classical variables such as sex, pain, physical activity, and individual psychosocial comorbidities (fatigue, anxiety, depression) did not remain significant in the multivariable model. This suggests these variables act primarily through their impact on central sensitization and sleep quality.

**Figure 1:**
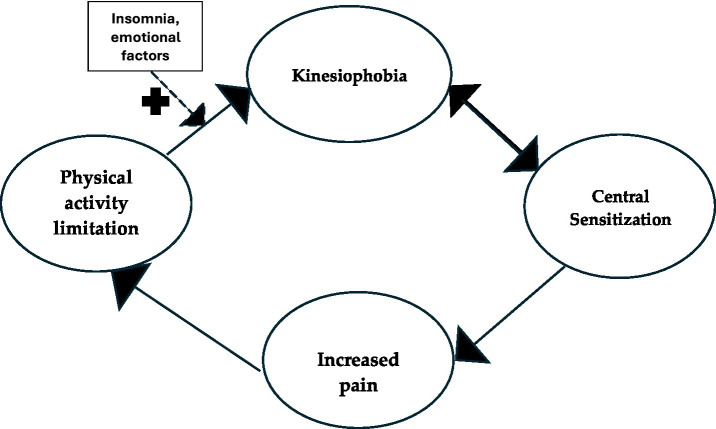
Hypothetical schema of kinesiophobia causes/consequences in PsA.

This model is consistent with the Fear-Avoidance Model (FAM) proposed by Vlaeyen and Linton, which establishes that catastrophic pain interpretation leads to movement fear, followed by avoidance, which paradoxically intensifies chronic pain and disability (7, 8). Following this premise and in accordance with our results, we developed the following schema synthesizing the process (Figure 1).

From this study, clinical implications emerge: given that nearly half of PsA patients may experience kinesiophobia and given its association with greater disease activity, routine screening in standard PsA patient evaluation should be considered. The TSK-11 is a brief (11-item), Spanish-validated, clinically practical instrument ideally positioned for clinical practice (22). Since kinesiophobia associates with multiple clinical variables (psychosocial comorbidities, reduced physical activity, limited functionality), interventions in these patients must be multifactorial.

Our work presents several limitations. First, the cross-sectional design did not permit causality establishment. It is impossible to determine whether kinesiophobia is cause or consequence of reduced physical activity, greater central sensitization, or psychosocial comorbidities. Furthermore, all patients were recruited from a single center, limiting generalizability to other geographic populations and healthcare systems. The multivariable model explained only 20% of kinesiophobia variance, which we interpret not as a limitation but as a meaningful finding: kinesiophobia is driven by biopsychosocial mechanisms that extend beyond pain severity, inflammatory activity, and standard comorbidity assessments. This observation underscores the complex, multidetermined nature of kinesiophobia and highlights the need for more granular assessment of psychological constructs (self-efficacy, resilience, trauma history, catastrophizing beliefs) in future research. Another limitation is the absence of a non-clinical comparison group. Our analyses contrasted patients with and without clinically significant kinesiophobia according to a validated cut-off, but we did not include a reference sample from the general population. Given that kinesiophobia has been described even among individuals without chronic disease, future studies should incorporate population-based control groups to better contextualize the prevalence and severity of kinesiophobia in PsA (48). Moreover, we must acknowledge that, due to the cross-sectional nature of the study, the assessment of the variables may have coincided with a disease flare, which could have introduced residual bias. Additionally, the TSK-11 > 26 cutoff used to define clinically significant kinesiophobia was derived from severity categories established in chronic musculoskeletal pain populations (23) and has not undergone specific validation in PsA. Although this threshold has been consistently applied in recent studies of inflammatory rheumatic diseases — including rheumatoid arthritis and gout —, facilitating cross-disease comparability, it remains a generic chronic pain threshold rather than a PsA-specific value. It is plausible that the optimal cutoff point for identifying clinically significant kinesiophobia in PsA may differ due to the unique clinical phenotype of this disease, which includes the coexistence of peripheral articular, axial, entheseal, and cutaneous manifestations, which may differentially modulate fear-of-movement responses. Future studies should explore PsA-specific cutoff values through receiver operating characteristic (ROC) curve analyses.

As strengths, to our knowledge, this is the first study specifically examining kinesiophobia in PsA with multiple domains systematically evaluated: disease activity, functionality, psychosocial variables, central sensitization, sleep, and physical activity.

## 5 Conclusion

Kinesiophobia is a clinically relevant phenomenon in PsA, present in 45.5% of patients and associated with greater central sensitization, sleep disturbance, increased disease activity, limited functionality, and psychosocial comorbidities. Kinesiophobia functions as an amplifier of pain and disability, operating through mechanisms of central sensitization and fear-avoidance that are largely independent of objective inflammatory burden. Unlike other classical clinical variables, central sensitization and sleep quality emerged as significant independent determinants in multivariable analysis.

The possible bidirectional relationship between kinesiophobia and central sensitization suggests that interventions targeting both domains—pharmacological (inflammation control optimization, psychopharmacological consideration) and psychosocial (cognitive-behavioral therapy, supervised exercise, sleep optimization)—may prove more effective than unidimensional approaches (48, 49). Recognizing kinesiophobia as a relevant clinical complication in PsA opens opportunities for integrated interventions optimizing not only inflammatory control but also functionality, quality of life, and psychosocial well-being in affected patients, particularly those with perceived disease burden exceeding objective inflammatory markers.

## Data Availability

The raw data supporting the conclusions of this article will be made available by the authors, without undue reservation.
